# Somatostatin-Expressing Interneurons Form Axonal Projections to the Contralateral Hippocampus

**DOI:** 10.3389/fncir.2019.00056

**Published:** 2019-08-23

**Authors:** Mark D. Eyre, Marlene Bartos

**Affiliations:** Medical Faculty, Institute for Physiology I, Systemic and Cellular Neurophysiology, University of Freiburg, Freiburg, Germany

**Keywords:** GABA, interneuron, G-deleted rabies, optophysiology, stereology, long-range, somatostatin, dentate gyrus

## Abstract

Conscious memories are critically dependent upon bilateral hippocampal formation, and interhemispheric commissural projections made by mossy cells and CA3 pyramidal cells. GABAergic interneurons also make long-range axonal projections, but little is known regarding their commissural, inter-hippocampal connections. We used retrograde and adeno-associated viral tracing, immunofluorescence and electron microscopy, and *in vitro* optogenetics to assess contralateral projections of neurochemically defined interneuron classes. We found that contralateral-projecting interneurons were 24-fold less common compared to hilar mossy cells, and mostly consisted of somatostatin- and parvalbumin-expressing types. Somatostatin-expressing cells made denser contralateral axonal projections than parvalbumin-expressing cells, although this was typically 10-fold less than the ipsilateral projection density. Somatostatin-expressing cells displayed a topographic-like innervation according to the location of their somata, whereas parvalbumin-expressing cells mostly innervated CA1. In the dentate gyrus molecular layer, commissural interneuron post-synaptic targets were predominantly putative granule cell apical dendrites. In the hilus, varicosities in close vicinity to various interneuron subtypes, as well as mossy cells, were observed, but most contralateral axon varicosities had no adjacent immunolabeled structure. Due to the relative sparsity of the connection and the likely distal dendritic location of their synapses, commissural projections made by interneurons were found to be weak. We postulate that these projections may become functionally active upon intense network activity during tasks requiring increased memory processing.

## Introduction

The hippocampus plays a key role in learning and memory of contexts and events. Information is processed via the classical canonical tri-synaptic path comprising the dentate gyrus (DG) as the input gate of the hippocampus, in which information is encoded by granule cells (GCs) and transmitted via their ‘mossy fibers’ to CA3 pyramidal cells (PCs), and from there via Schaffer collaterals to CA1 PCs. Similar to these strong glutamatergic connections along this tri-synaptic pathway, projections between the two hippocampi are known to exist. These are primarily made by GCs, mossy cells (MCs) located in the hilar area (between the CA3c region and the GC layer of the DG) and CA3c-PCs (Swanson et al., 1981; Ribak et al., 1985, 1986). Although the glutamatergic local pathways within the hippocampal circuitry and between the ipsi- and contralateral hippocampus have been extensively documented (Zola-Morgan and Squire, 1993; Squire and Wixted, 2011), very little attention has been paid to inter-hippocampal connections arising from GABAergic inhibitory cells. Many studies recorded from cells from a single hemisphere *in vivo* or from cells in acute slice preparations, which allow examination of connections between the subfields within a single hippocampus, but do not preserve contralateral connections. Indeed, the lateralization of hippocampal function and communication between the two hemispheres, even on a general level, has been examined in very few studies (Czéh et al., 1998; de Hoz et al., 2005; Klur et al., 2009), leaving questions on the nature of commissurally projecting GABAergic cells open.

GABAergic interneurons are a highly diverse population of cells implicated in many microcircuit functions, particularly their control of PC computation (Han et al., 1993; Freund and Buzsáki, 1996; Houser, 2007; Hosp et al., 2014; Urban-Ciecko and Barth, 2016). Although classically characterized as local circuit neurons, some GABAergic cells have been previously described in a variety of brain areas that make longer-range axonal projections to non-adjacent brain regions. Different subclasses of interneurons in CA1 are known to project axons to a variety of targets, such as CA3 and the DG (Sik et al., 1994; Fuentealba et al., 2010). Interneurons in CA1 expressing the muscarinic M2 receptor are known to project to the retrosplenial cortex (Miyashita and Rockland, 2007), whereas CA1 interneurons expressing Enkephalin (Fuentealba et al., 2008) or Vasoactive Intestinal Polypeptide (Francavilla et al., 2018) project to the subiculum. Interneurons residing in the DG outer molecular layer have also been reported that project to the subiculum (Ceranik et al., 1997). In the DG hilus, somatostatin (SOM)-expressing interneurons (SOMIs) densely innerve the ipsilateral hilar area, but also send axon collaterals to the medial septum and form synaptic contacts onto glutamatergic, cholinergic and GABAergic cells (Jinno et al., 2007; Yuan et al., 2017). Similarly, SOMIs in CA1 connect with the medial septum (Tóth et al., 1993; Gulyas et al., 2003; Takács et al., 2008). Moreover, SOMIs in the entorhinal cortex project to the superficial molecular layer of the DG and hippocampal SOMIs project to the entorhinal cortex (Melzer et al., 2012). However, potential interhemispheric interactions remain mostly uninvestigated (see Léránth and Frotscher, 1987; Zappone and Sloviter, 2001). In this study, we used retrograde tracers and viral expression of fluorescent markers in combination with transgenic mice to provide evidence for inter-hippocampal connections made by SOM- and parvalbumin (PV) -expressing GABAergic cells.

## Materials and Methods

### Animals and Stereotaxic Injections

Male and female mice of the following strains were used for experiments: wild type C567Black6J mice (henceforth WT mice), Jax Mice Stock Number: 000664 | Black 6; mice heterozygous for the insertion of green fluorescent protein (GFP) under the control of the GAD67 gene (GAD67-GFP mice), Tamamaki et al. (2003); mice homozygous for the insertion of cre-recombinase under the control of either the somatostatin, parvalbumin or GAD65 gene (SOM-cre, Jax Mice Stock Number: 013044 | Sst-IRES-Cre; PV-cre, Jax Mice Stock Number: 017320 | B6 PV cre; GAD2-cre, Jax Mice Stock Number: 010802 | Gad2-IRES-Cre, respectively); mice from these cre-expressing lines crossed with the Ai9-tdTomato cre-reporter line, Jax Mice Stock Number: 007909 | Ai9 or Ai9(RCL-tdT). All experimental procedures were performed in accordance with the ethical guidelines of the University of Freiburg and the State of Baden-Württemberg (Regierungspräsidium Freiburg), Germany. Mice were anesthetized with Isoflurane (3% induction, 1.75% maintenance) and mounted on a Kopf stereotaxic frame. Animals were injected with an adeno-associated virus (AAV) expressing GFP under the CAG promoter in a cre-dependent manner (AAV2/1-CAG-FLEX-GFP; University of Pennsylvania Vector Core, Philadelphia, PA, United States) or with fluorescent microspheres (Retrobeads; Lumafluor Inc.).^1^ In some animals, both were injected, either separately, one in each hemisphere, or unilaterally as a 1:1 mixture at the same site. We also performed retrograde tracing with fluorescently conjugated Cholera Toxin Subunit B, and found a pattern of labeling similar to that seen with Retrobeads, i.e., almost exclusively in mossy cells. For monosynaptic rabies tracing experiments we injected an AAV expressing GFP, the avian TVA receptor and the Rabies protein G in a cre-dependent manner (AAV2/8-EF1a-FLEX-GFP-T2A-hTVA-E2A-hB19G). Twenty-two days later, an avian EnvA-coated, G-deleted Rabies virus expressing mCherry was injected at the same co-ordinates, and animals were sacrificed 17 days later. For the functional identification of postsynaptic targets using *in vitro* optogenetics, an AAV expressing Channelrhodopsin2 (ChR2) with a YFP tag (AAV2/1-CAG-FLEX-ChR2-YFP) was injected. The stereotaxic co-ordinates and parameters used in this study can be found in Supplementary Table 1.

### Immunohistochemical Processing for Fluorescent and Electron Microscopy

Between 13 and 27 days post-injection, animals were anesthetized and transcardially perfused with 0.9% NaCl for 1 min followed by 4% Paraformaldehyde in 0.1M Phosphate buffer (PB) for 15 min at a flow rate of 7 ml per minute using a peristaltic pump. Brains were removed from the skull, washed in PB and cut into 70 or 100 μm-thick coronal sections using a vibratome (VTS1000, Leica). Sections were washed three times in Tris-buffered saline (TBS), blocked in 10% Normal Goat Serum (NGS) for 60 min and then incubated overnight at 23°C in a primary antibody solution diluted in TBS containing 2% NGS and 0.5% Triton-X 100 (TBST). After three washes in TBS, sections were incubated in a solution of secondary antibody diluted in TBST for 2 h at 23°C, then washed three times, and mounted in Mowiol. Sections for cellular and axonal density quantification were imaged using a Zeiss 710 Laser-scanning confocal microscope equipped with a 20× (0.8 NA) objective and with either 0.7× or 4× digital zoom. Images of Retrobead labeling were acquired using a 63× (1.4 NA) objective and 0.7× digital zoom. The following primary antisera were used: rabbit anti-somatostatin (1:1000, Peninsula Labs), rabbit anti-parvalbumin (1:1000, Swant), rabbit anti-calretinin (1:5000, Swant), guinea-pig anti-calbindin (1:500, Synaptic Systems), rabbit anti-cholecystokinin (1:500, Frontier Institute), mouse anti-GluA2 (1:1000, NeuroMab), guinea-pig anti-neuropeptide-Y (1:500, Abcam), rabbit anti-Kv3.1b (1:500, Alomone). The following secondary antisera were used: Alexa Fluor 555-conjugated- or Alexa Fluor 647-conjugated- goat anti-rabbit, goat anti-guinea-pig or goat anti-mouse (all 1:500, Invitrogen). In some cases, a Nissl stain (DAPI) was used to aid the identification of immunolabeled somata and different hippocampal strata.

For the quantification of cell or axon densities, a series of Z-stacks were acquired in a systematic way with a random start location from each sub-field of the DG and hippocampus. In each animal, for at least three sections each, three fields of view were imaged that encompassed the medial-to-lateral extent of each region. Each field of view was acquired using a 20x air objective with a 4x digital zoom. Each field of view was 106 μm × 106 μm, although in some cases smaller fields of view were used when axon or cell density was high, particularly for fields of view in the hemisphere ipsilateral to the injection. The z depth was typically 30 slices at 1 μm spacing, but ranged between 16 and 51 slices. For axons, the mean sampled volume was 540085 ± 248582 μm^3^ and ranged between 89267 and 1151918 μm^3^ for contralateral stacks, and was 283400 ± 215029 μm^3^ and ranged between 13070 and 1262255 μm^3^ for ipsilateral stacks. For labeled cells, the mean sampled volume was 21897387 ± 38433454 μm^3^ and ranged between 1227983 and 175041447 μm^3^. We did not correct for shrinkage. Data are reported as cellular density or axonal length density. The CA2 area and the very medial part of CA1 were avoided because the borders of these areas could not be determined with reliable certainty using morphology alone, and we had no spare color channels for additional immunofluorescent markers. An optical disector approach was applied to the Z-stacks, treating each one as a sampling volume, either counting the number of cells manually or measuring the length of GFP-labeled axon within the volume using the Image-J Simple Neurite Tracer plug-in. For the quantification of Retrobead labeling colocalization with immunofluorescent labeling, 1 or 2 sections were imaged in 2 non-overlapping locations encompassing almost the entire hilus (close to the hilus tip where to two GC blades meet, and close to the area adjacent to CA3c) for each immunolabeling condition.

Some vibratome sections were instead immersed in a solution of 12.5% sucrose and 5% glycerol in PB for 60 min, and then 25% sucrose and 10% glycerol overnight at 4°C before being freeze-thawed over liquid nitrogen three times. After thorough washing in PB, sections were incubated in 1% H_2_O_2_ for 10 min, washed three times in TBS, blocked with 10% NGS in TBS for 60 min and then incubated in rabbit anti-GFP (1:500, Invitrogen) in TBS containing 2% NGS overnight at 23°C. After three washes in TBS, sections were incubated in a solution of biotinylated goat anti-rabbit secondary antibody diluted 1:50 in TBS with 2% NGS overnight at 4°C. After three washes in TBS, sections were incubated in an Avidin-Biotin complex (1:100, Elite ABC) for 120 min, washed three times in Tris Buffer (TB), incubated in a 0.05% solution of DiAminoBenzidine (DAB) in TB for 20 min, and then H_2_O_2_ was added (final concentration 0.6%). After 10 min of reaction, sections were washed three times and then reacted with 1% OsO_4_ in PB for 20 min, dehydrated in an ascending concentration series of ethanol, finishing with a step in propylene oxide, and were then embedded in Durcupan resin on microscope slides and cured at 60°C for 24 h. Tissue blocks were dissected and re-embedded in Durcupan, sectioned at 70 nm thickness using an ultra-microtome (Ultracut, Leica Microsystems) and imaged using a Zeiss LEO906 electron microscope.

### Electrophysiology

For whole-cell patch clamp recordings from a cohort of mice with unilateral injection of Retrobeads into the dorsal hilus, the post-injection survival time was between 1 and 8 days. For experiments using the ChR2-expressing virus, the post-injection survival time was between 14 and 25 days. In all cases, mice were anesthetized with isoflurane and decapitated. The brain was removed from the skull and placed into a sucrose-based, ice-cold solution (SUCROSE) containing the following (in mM): 230 sucrose, 2.5 KCl, 25 glucose, 1.25 NaH2PO_4_, 24 NaHCO_3_, 4 MgCl_2_, and 0.5 CaCl_2_, bubbled continuously with 95% O_2_ and 5% CO_2_, resulting in a pH of 7.4. In all cases, coronal slices from the dorsal hippocampus were cut at a thickness of 300 μm with a Vibratome (VT1200S; Leica) and were stored in artificial cerebrospinal fluid (ACSF) containing the following (in mM): 125 NaCl, 2.5 KCl, 25 glucose, 1.25 NaH_2_PO_4_, 25 NaHCO_3_, 1 MgCl_2_, and 2 CaCl_2_, bubbled continuously with 95% O_2_ and 5% CO_2_, (pH = 7.4). After a 30 min recovery period at 34°C, slices were further incubated at room temperature (23°C) until they were transferred to the recording chamber. For some experiments, to facilitate cell survival in slices from older animals, injected mice were anesthetized and transcardially perfused with 30 ml of ice-cold sucrose solution. Slices were cut in ice-cold-sucrose, as above, but were then transferred to a recovery solution (NMDG) containing the following (in mM): 93 *N*-methyl-D-glucamine, 2.5 KCl, 25 glucose, 1.25 NaH2PO_4_, 24 NaHCO_3_, 20 HEPES, 5 Sodium Ascorbate, 2 Thiourea, 3 Sodium pyruvate, 10 MgCl_2_, 0.5 CaCl_2_ and 12 *N*-acetyl-L-cysteine, bubbled continuously with 95% O_2_ and 5% CO_2_, (pH = 7.4). After a 10 min recovery period at 34°C in this NMDG solution, slices were transferred to a similar solution except that the *N*-methyl-D-glucamine was replaced with 92 mM NaCl (RECOVERY). Slices were held in this recovery solution at room temperature until they were transferred to the recording chamber, and were recorded in ACSF.

Somatic whole-cell recordings were performed at 23°C using infrared differential interference contrast on a Zeiss Examiner Microscope with a 40x water-immersion objective. Recording pipettes (wall thickness: 0.5 mm; inner diameter: 1 mm) were pulled from borosilicate glass tubing (Hilgenberg, Germany; Flaming-Brown P-97 puller, Sutter Instruments, United States), filled with a solution containing (in mM): 110 K-Gluconate, 40 KCl, 10 HEPES, 7 Phosphocreatine, 2 MgCl_2_, 2 Na_2_ATP, 0.5 Na-GTP, 0.025 EGTA and 0.0054 biocytin (pH = 7.33, 270–290 mOsm). Signals were filtered at 5–10 kHz and digitized at 20–40 kHz with a Power1401 laboratory interface (Cambridge Electronic Design, United Kingdom). All recordings were performed with a MultiClamp 700B amplifier (Molecular Devices), and data were digitized on-line at 20 kHz. ChR2 was activated by application of blue light pulses (473 nm; 5 ms, 0.2 Hz, full field illumination; CoolLED system, United Kingdom). Stimulus-generation and data acquisition, including LED pulse generation, were performed with a custom-made Igor-based program (FPulse, courtesy of U. Froebe, University of Freiburg, Freiburg, Germany).

### Statistical Data Analysis and Presentation

All data are expressed as the mean ± standard deviation. All statistical comparisons were made with SigmaPlot 11.0.0.75 (SyStat Software, Inc.). A Shapiro–Wilk test for Normality of distribution was applied to the cell and axon length density data, and because some were not normally distributed, a Spearman Rank Order Correlation was used to assess the relationship between the datasets. Data for somatic labeling were pooled across sub-regions in order to avoid the strong influence of zero-value datasets for sub-regions without somatic labeling (e.g., CA1 *stratum radiatum*; see also Table 1). Differences were considered different at *p* ≤ 0.05. Data reported in Table 1 are mean values ± standard deviation from the number of animal replicates indicated. In order to present the data in a visually meaningful way, for each region, the value divided by the highest value among all regions in the entire dataset was calculated, the square root was taken, and then the value was expressed as a percentage. This percentage was used to set the transparency of the color for that region in the schematic; pure red was used for axon length densities; pure blue, and a different scaling, was used for soma densities.

**TABLE 1:** Numerical values (mean ± standard deviation) of AAV-Flex-GFP-labeled number of somata (cells per mm^3^) and axonal length (mm per mm^3^) presented graphically in Figure 2.

**Genotype**	**GAD2-cre**	**SOM-cre**	**SOM-cre**	**SOM-cre**	**SOM-cre**	**PV-cre**
Category	hippocampus	hippocampus	hilus and CA3	hilus	CA1	hilus and CA3
*n*	3	3	4	4	4	4
**Ipsilateral labeled cell density: cell somata/mm^3^**						
CA1 slm	4995 ± 4468	0 ± 0	0 ± 0	0 ± 0	0 ± 0	0 ± 0
CA1 sr	1103 ± 974	19 ± 33	0 ± 0	0 ± 0	12 ± 25	0 ± 0
CA1 sp	2799 ± 4848	470 ± 425	0 ± 0	0 ± 0	682 ± 802	0 ± 0
CA1 so	324 ± 561	2679 ± 1716	0 ± 0	0 ± 0	4322 ± 3550	0 ± 0
CA3 so	3209 ± 2841	2157 ± 1585	1977 ± 699	0 ± 0	0 ± 0	196 ± 174
CA3 sp	1257 ± 1091	112191 ± 13552	2757 ± 1369	380 ± 760	0 ± 0	2660 ± 2270
CA3 sr	392 ± 496	534 ± 204	1033 ± 929	25 ± 51	0 ± 0	300 ± 257
DG hilus	27441 ± 3654	5104 ± 2399	5369 ± 2292	11316 ± 10132	0 ± 0	3369 ± 4334
DG GCL	6292 ± 3272	207 ± 358	28 ± 56	0 ± 0	0 ± 0	3149 ± 1480
DG ML	861 ± 530	0 ± 0	0 ± 0	0 ± 0	0 ± 0	16 ± 32
**Ipsilateral axon density: mm of axon/mm^3^**						
CA1 slm	6605 ± 3717	34873 ± 22808	985 ± 897	1135 ± 1334	24683 ± 33313	479 ± 959
CA1 sr	9264 ± 14535	17807 ± 17481	1398 ± 588	1522 ± 2629	7875 ± 4702	1044 ± 1621
CA1 so	6535 ± 10987	16278 ± 23234	576 ± 434	1527 ± 2955	7976 ± 3865	595 ± 1017
CA3 sr	6911 ± 9013	14556 ± 12502	22843 ± 12522	463 ± 508	2054 ± 3382	2191 ± 1111
CA3 so	2654 ± 4529	12726 ± 18058	23500 ± 17405	339 ± 246	409 ± 644	1984 ± 1439
DG infra	23855 ± 8650	31796 ± 30678	34674 ± 29970	41453 ± 15393	122 ± 164	3221 ± 2146
DG supra	33105 ± 16050	30884 ± 21990	27114 ± 24566	38536 ± 38571	498 ± 728	4097 ± 1013
DG hilus	7683 ± 2716	13319 ± 14661	18457 ± 13965	18856 ± 25792	31 ± 42	7817 ± 2554
**Contralateral axon density: mm of axon/mm^3^**						
CA1 slm	955 ± 629	330 ± 350	376 ± 345	115 ± 130	37 ± 75	138 ± 276
CA1 sr	1629 ± 1030	5919 ± 5064	974 ± 1440	687 ± 775	939 ± 983	362 ± 464
CA1 so	1373 ± 1227	3329 ± 5315	536 ± 627	72 ± 62	737 ± 692	46 ± 92
CA3 sr	243 ± 237	2646 ± 3438	1456 ± 988	118 ± 167	399 ± 798	79 ± 94
CA3 so	933 ± 432	4124 ± 6180	2263 ± 2496	312 ± 548	476 ± 952	0 ± 0
DG infra	2658 ± 1098	2346 ± 2184	1284 ± 797	3041 ± 4048	34 ± 69	49 ± 97
DG supra	1838 ± 647	3580 ± 2391	1704 ± 1425	1682 ± 1953	183 ± 367	196 ± 240
DG hilus	1132 ± 763	1267 ± 892	462 ± 433	532 ± 416	297 ± 594	0 ± 0

For Retrobead (Ret) labeling experiments, we computed the densities of different cell populations using the optical disector, as above. For immunolabeling using SOM, PV and CB, we considered these markers to be specific for interneurons. The total interneuron population in these three experiments was defined as the cell group expressing GAD67-GFP plus the group expressing the particular interneuron marker used plus any cells labeled by both. We then counted the total projecting interneuron population as those Ret^+^ cells also labeled by the marker (GAD67-GFP-expressing plus not-GAD67-GFP-expressing). For the mossy cell population, we performed two immunolabeling experiments for CCK and GluA2, and included cells that were Ret^+^ and immunolabeled (projecting population) plus cells that were just immunolabeled (non-projecting population). For the GluA2 immunolabeling, we detected many cells that were not Ret^+^, suggesting that this marker is not as specific for mossy cells as CCK. Interestingly, we also note that only 42.6% (2503 cells/mm^3^) of the entire hilar SOMI population expressed GAD67, indicating that the remaining 57.4% (3288 cells/mm^3^) may express GAD65 instead.

### Rabies Tracing

Monosynaptic retrograde Rabies virus tracing splits the rabies virus genome into two components that must combine in order to express a transmission competent, but replication deficient, rabies virus. A sparse population of cells are infected with an AAV expressing a GFP marker, the Rabies coat protein G and an avian receptor pseudotyped for Rabies infection, all in a cre-dependent manner. Subsequent infection by a correctly pseudotyped protein-G-deleted rabies virus with a mCherry marker allows the deficient rabies to become transmission competent and infect presynaptic neurons. Due to the spatial restriction of AAV expression, presynaptic neurons in distant brain regions will selectively express deficient Rabies and only mCherry, but will no longer be infectious. Unilateral AAV infection in the DG of GAD2-cre mice with AAV8-GFP-T2A-hTVA-E2A-hB19G induced a cre-dependent expression of GFP, Protein G and the hTVA receptor selectively in GAD2-cre-expressing cells. These GAD2-expressing cells were thus primed for Rabies infection. Twenty-two days later, animals were injected at the same co-ordinates with EnvA-RVdRG-mCherry, and animals were allowed to survive for a further 17 days. Both components of this monosynaptic Rabies tracing system were obtained from the Salk Institute for Biological Studies, 10010 N. Torrey Pines Rd, La Jolla, CA, 92037. After transcardial perfusion, brains were sliced on a vibratome and fluorescent immunocytochemistry was performed against several interneuron and mossy cell-specific markers, as above. Cells infected by both viruses therefore expressed GFP and mCherry, and were classified as ‘starter cells’ that produced trans-synaptic retrograde transmission-competent Rabies virus particles. These particles infected presynaptic cells in a cre- and TVA-independent manner, and these presynaptic cells therefore also expressed mCherry. Confocal images of mCherry and immunofluorescently labeled cells in the hippocampus contralateral to the injection site were acquired for qualitative analysis. Injections were also performed in SOM-cre and PV-cre mice.

## Results

### Contralaterally Projecting Hippocampal GABAergic Interneurons

**FIGURE 1:**
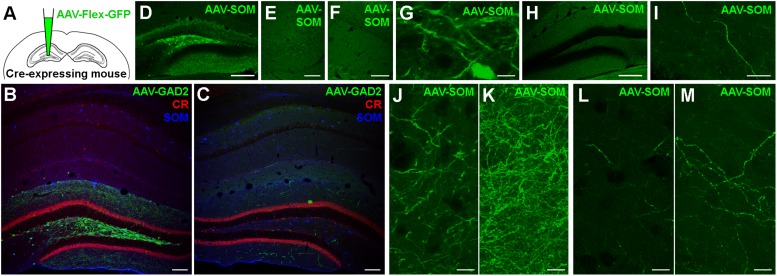
Somatostatin-expressing hilar interneurons make axonal projections to the contralateral hippocampus. **(A)** Schematic of the experimental approach. **(B)** Localized expression of AAV1-FLEX-CAG-GFP in the injected brain hemisphere after stereotaxic injection into the dentate gyrus hilus region of a GAD2-cre mouse. Coronal section. Single confocal plane. Scale bar = 100 μm. **(C)** Contralateral hippocampus of the same section shown in **(B)**. Note the lack of somatic labeling, but labeling of axons, particularly in the dentate molecular layers. Single confocal plane. Scale bar = 100 μm. **(D)** Localized expression of AAV1-FLEX-CAG-GFP in the injected brain hemisphere after stereotaxic injection into the dentate gyrus hilus region of a SOM-cre mouse. Coronal section. Single confocal plane. Scale bar = 200 μm. **(E–G)** GFP was expressed in somata of the ipsilateral hilus **(G)**, but not in CA3 **(E)** or CA1 **(F)**. Single confocal sections. Scale bars = 10, 50, and 50 μm. **(H)** Low magnification image of the contralateral hemisphere of the same section shown in **(D)**, indicating a lack of somatic GFP. Single confocal section. Scale bar = 200 μm. **(I)** High magnification image of a GFP-labeled axon in the contralateral hilar region. Maximum intensity projection of 28 planes (1 μm spacing). Scale bar = 20 μm. **(J,K)** Viral GFP-expressing axons in the ipsilateral Infragranular molecular layer, either as a single confocal section **(J)** or the same field of view as a maximum intensity projection of 25 planes (**K**, 1 μm spacing). Scale bars = 10 μm. **(L,M)** Viral GFP-expressing axons in the contralateral Infragranular molecular layer, either as a single confocal section **(L)** or the same field of view as a maximum intensity projection of 25 planes (**M**, 1 μm spacing). Scale bars = 10 μm.

To test whether GABAergic cells project to the hippocampus of the contralateral hemisphere, we used mice expressing Cre-recombinase under the control of the GAD2 promoter (GAD2-cre mice; see section Materials and Methods for details of mouse lines used) and stereotaxically injected an AAV expressing GFP in a cre-recombinase-dependent manner unilaterally into the DG (Figure 1A and Supplementary Table 1). Eighteen days after injection, we observed somatic GFP expression in GABAergic interneurons of the ipsilateral hippocampus (Figure 1B). Most of the labeled somata were located in the hilus, with a few scattered cells in the molecular layer of the DG and in the various layers of CA1-3. The majority of axonal arborizations of the labeled cells were distributed in the ipsilateral hilus, the granule cell layer (gcl) and molecular layer, with weaker axonal distributions in various layers of CA1-3. In the contralateral hippocampus, somatic GFP-expression was not observed, ruling out retrograde and trans-synaptic anterograde labeling by the AAV. Interestingly, numerous axon-like processes were found in all contralateral hippocampal regions, albeit at markedly lower densities than their ipsilateral counterparts (Figure 1C and Supplementary Figure 1). Thus, our data indicate contralaterally projecting GABAergic fibers originating in the ipsilateral DG and hilus.

### Ipsilateral SOMIs Innervate the Contralateral Hippocampus

Previous studies showed that among GABAergic neuron types, SOMIs in particular form long-range projections to cortical and subcortical areas (Alonso and Köhler, 1982; Ferraguti et al., 2005; Takács et al., 2008; Yuan et al., 2017). We therefore asked whether SOMIs might contribute to the population of contralaterally projecting GABAergic cells by injecting AAV-FLEX-GFP into different hippocampal subregions of SOM-cre mice and examined the distribution of contralaterally projecting GFP-positive fibers (Figure 1D). Infection of the entire ipsilateral hippocampus resulted in GFP-expressing somata in the ipsilateral hilus, stratum oriens and alveus of CA1-3 regions, consistent with the expected distribution patterns of SOMIs in the rodent hippocampus (Freund and Buzsáki, 1996; Houser, 2007; Hosp et al., 2014). Injections restricted to the DG-hilus resulted in GFP-labeling of medium-sized, elongated somata with moderately spiny dendrites in the hilar area (Figure 1G). GFP-labeled axons in the ipsilateral hemisphere were particularly observed in the molecular layer (Figures 1J,K) and at lower densities in the hilus and the gcl (Figure 1D), consistent with the axonal distribution of previously identified SOM-expressing hilar perforant path-associated interneurons (HIPPs; Hosp et al., 2014; Yuan et al., 2017), but no labeling was observed in the ipsilateral CA3 or CA1 (Figures 1E,F). Somatic GFP labeling contralateral to the injected hemisphere was not observed in any mice (Figure 1H). However, sparse GFP-positive axons were present in the contralateral hilus (Figure 1I) and molecular layer (Figures 1L,M). The contralateral axons were varicose and of similar appearance and caliber as ipsilateral axons, but were much more sparse, only occasionally bifurcating, and appeared to meander in various orientations for long distances. These ramification patterns could not be easily categorized as belonging to any previously identified interneuron type (Freund and Buzsáki, 1996; Houser, 2007; Hosp et al., 2014).

**FIGURE 2:**
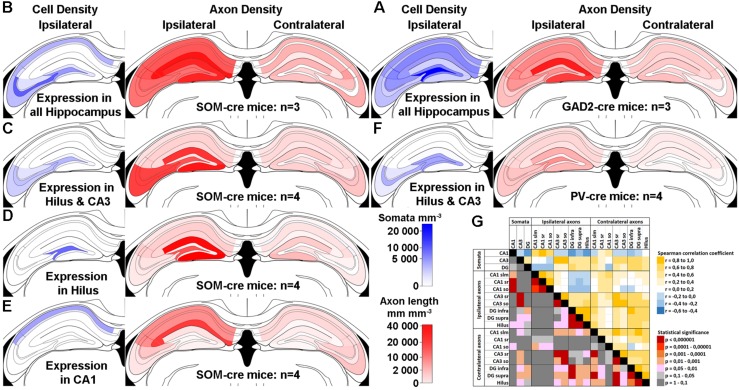
Contralateral projections mirror the target areas of their ipsilateral counterparts, but are less dense. **(A–F)** Standardized schematic quantitative colormap plots of average GFP-labeled cell density (blue) and axon length per unit volume (red) measured following GFP expression upon stereotaxic AAV injection into the dorsal hippocampus. **(A)** Infection of the entire ipsilateral hippocampus in GAD2-cre mice, with widespread Ipsi- and Contralateral axonal arborization. **(B–E)** Localized virus injections infecting the entire hippocampus **(B)**, hilus and CA3 **(C)**, hilus only **(D)** or CA1 only **(E)** of SOM-cre mice. **(F)** Infection of the hilus and CA3 region in PV-cre mice. In all cases, color intensity is plotted on the same sub-linear scale so that the smaller contralateral values are visible. Infection location(s), genotype and number of animals are indicated. Average measured values are listed in Table 1 for numerical comparison. The CA2 area was not distinguished and is grouped with CA1. Images show the injected ipsilateral hemisphere on the left by convention, although unilateral injections were performed in either hemisphere. We did not observe any differences between injections into left or right hemispheres. No contralateral somatic labeling was seen in any hippocampal regions in any of the injected mice. **(G)** Color-coded Spearman Rank Order correlation coefficients and associated statistical significance for comparisons between different sub-regions using all the data from SOM-cre mice plotted in **(B–E)**.

Do the remaining hippocampal sub-regions also show contralaterally projecting SOMIs? To address this question, we grouped our ipsilateral infections into several cohorts according to the regions of somatic GFP expression (Figure 2). We quantified the axonal labeling by measuring the length of axons within a given sample volume using the Image J Simple Neurite Tracer plug-in (see section Materials and Methods). Confocal Z-stacks of regions of interest were acquired in a systematic random manner for sub-fields of the DG and CA1-3 regions from several sections in each animal (see section Materials and Methods). An estimate of labeled axon density (length/unit volume) was calculated and the data (Table 1) were visualized for the dorsal hippocampus using a non-linear color scale (Figure 2). The density of GFP-labeled somata was estimated for the hippocampal regions using the optical disector technique (Image J; see section Materials and Methods; West et al., 1991).

We observed that, first, the pattern of labeling across brains in SOM-Cre mice was similar to the one in GAD2-cre mice (Figure 2A vs. Figure 2B). However, closer inspection showed that there were differences in the densities of the axons, which may be explained, in part, by the different extents of somatic infection, and the broader range of interneuron types likely to be infected in the GAD2-cre mice. Second, the axon density in the contralateral hemisphere (averaged across the entire hippocampus) was 9.4 ± 7.7% of the ipsilateral measurement (14040 ± 12242 mm/mm^3^ vs. 1320 ± 940 mm/mm^3^). Third, when comparing infections of different extent, our data indicated a topographic organization of the contralateral SOMI-mediated projection. SOMI-GFP expression restricted to the DG-hilus and CA3 area resulted in defined axon labeling in the corresponding contralateral regions (Figure 2C). Interestingly, GFP labeling in the contralateral CA1-2 region was mostly absent. Moreover, SOM-GFP expression restricted to the ipsilateral DG-hilus resulted in a labeling largely focused on the contralateral DG, particularly the molecular layer (Figure 2D). Similarly, AAV injection restricted to CA1 resulted in contralateral GFP-positive fibers confined to CA1 (Figure 2E). Thus, SOMIs project to the contralateral hippocampus in a topographic, organized manner, whereby SOMIs located in a particular hippocampal subfield seem to preferentially target the corresponding area on the contralateral side.

Previous studies proposed that hilar parvalbumin (PV)-expressing interneurons (PVIs) may project to contralateral hippocampal areas (Leranth and Frotscher, 1986). We therefore tested whether PVIs contribute to contralateral projections by injecting AAV-FLEX-GFP in PV-cre mice (Figure 2F). PV-GFP labeling restricted to somata in the DG and CA3 resulted in the labeling of contralaterally projecting axons, but the density was markedly lower than the one measured for SOMIs (109 ± 158 mm/mm^3^ for PV-GFP; 1132 ± 669 mm/mm^3^ for SOM-GFP; Figure 2F vs. Figure 2C). Moreover, contralateral PV-GFP axons were primarily located in CA1, rather than the contralateral DG and CA3 counterparts of the injection zone. Thus, in contrast to SOMIs, DG-PVIs seem to preferentially target the contralateral CA1 area. We were concerned that this difference might be caused by the same virus reacting differently in the different cre-expressing mouse lines. Although we did not have any suitable method for measuring the diffusion of virus upon stereotaxic injection, we were careful to use similar parameters in each mouse line. We observed ipsilateral somatic labeling that spanned multiple vibratome sections (up to several millimeters) along the dorsal-ventral and longitudinal axes of the hippocampus within the intended region of injection in all animals included in the study, and thus believe that the virus diffusion was similar in each case. We used fluorescent immunolabeling as a comparison for estimating the viral expression efficiency in a subset of animals, and found that most cells immunopositive for the interneuron marker in question were also labeled by virally expressed GFP (in SOM-cre mice, 78.3 ± 21.7% of SOM-immunoreactive cells were also AAV-FLEX-GFP-positive; in PV-cre mice, 86.0 ± 4.2% of PV-immunoreactive cells were also AAV-FLEX-GFP-positive). We selected cases where the immunolabeling was optimal, resulting in few cases of cells only expressing AAV-FLEX-GFP (3.2 ± 5.5% in SOM-cre mice; 3.9 ± 3.0% in PV-cre mice), in order to ensure that we reliably immunolabeled the entire interneuron subpopulation.

To quantify whether SOMI-mediated contralateral projections are potentially topographically organized, we performed a Spearman Rank Order Correlation analysis between the number of ipsilaterally labeled SOMI somata in each hippocampal region (pooled for all subregions) and the axon densities in the corresponding ipsi- and contralateral hippocampal layers (Figure 2G). Strong positive correlations (*r* ≥ 0.64, *p* ≤ 0.003) were observed between ipsilateral somatic and axonal labeling densities in many hippocampal sub-regions, and axons within each ipsilateral hippocampal sub-region correlated strongly with each other (for example, CA3 *stratum radiatum* vs. *stratum oriens*, *r* = 0.95, *p* < 0.0001). When comparing ipsilaterally labeled SOMI somata with contralateral axons, the axon density in the contralateral DG did not correlate with soma densities of SOMIs in the ipsilateral hilus (*r* ≤ 0.50, *p* ≥ 0.06). In contrast, axon densities in the contralateral *stratum oriens* strongly correlated with ipsilateral soma labeling for CA1 (*r* ≥ 0.53, *p* ≤ 0.05) and even more strongly for CA3 (*r* ≥ 0.70, *p* ≤ 0.033). Significant correlations were also observed between most ipsilateral and contralateral axon densities for the same region (*r* ≥ 0.58, *p* ≤ 0.05), except for CA1 *stratum lacunosum moleculare* (slm) and *radiatum* (*r* ≤ 0.51, *p* ≥ 0.05). Comparisons of contralateral axon densities in the different layers of each sub-region also correlated with each other in the DG and CA3 (*r* > 0.77, *p* < 0.003) but not in CA1 (*r* < 0.50, *p* > 0.05). Interestingly, very strong correlations were observed between axon labeling in contralateral CA1 slm vs. contralateral CA3 sr and DG areas (*r* > 0.70, *p* < 0.00001), as well as for contralateral CA3 sr vs. contralateral DG areas (*r* > 0.69, *p* < 0.004). Taken together, DG and CA1-3 SOMI contralateral projections appear to innervate the layers of the corresponding hippocampal sub-region, but there are also a few strong interdependent areas such as the CA1 slm, CA3 sr and the DG, suggesting some strong common contralateral (divergent) projections from an ipsilateral somatic origin.

### Contralateral Connections of the Ventral Hippocampus

We also made a series of injections into the ventral hippocampal areas, targeting the Hilus and the CA3 regions with injections of AAV-Flex-GFP. In contrast to injections in the dorsal hippocampus, we found that the contralateral projection from these ventral regions were either very weak or non-existent, despite similar ipsilateral axonal labeling (data not shown). Likewise, Ret and CTB injections labeled no interneurons in either the dorsal or the ventral contralateral hippocampus. We did observe neurons with very strong Ret or CTB labeling in the contralateral hilus after such injections, but these cells lacked immunoreactivity for either SOM or PV, and so we concluded that these were mossy cells. Together, these data are consistent with reports in the literature indicating that contralateral projections are made mainly by glutamatergic cell types in the dorsal hippocampal regions.

### Retrograde Tracing Confirms That Hilar SOMIs Innervate the Contralateral Hippocampus

**FIGURE 3:**
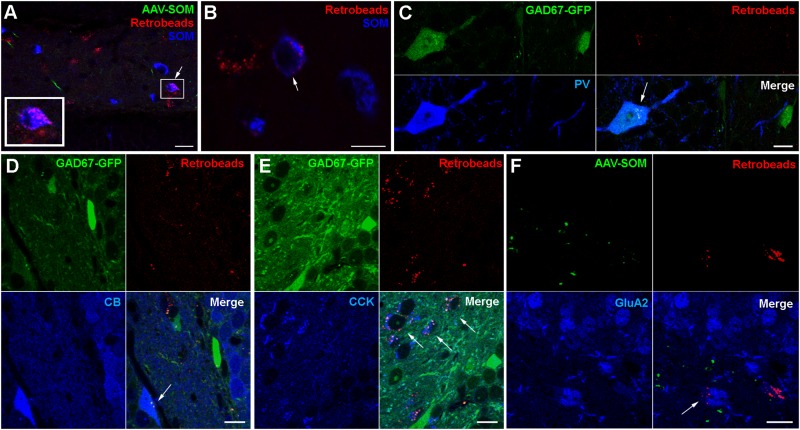
Retrograde tracing identifies populations of contralaterally projecting neurons that are immunoreactive for different neurochemical markers (arrows in all panels). **(A)** Retrobead labeling from the contralateral hilus (red) and immunolabeling for SOM (blue) reveal occasional dual-labeled cells (boxed region, shown enlarged at bottom left). Maximum intensity projection of 9 planes (1 μm spacing). Scale bar = 10 μm. **(B)** Another example showing a Retrobead-labeled cell (red, left), a SOM-immunoreactive cell (blue, right) and a soma labeled for both (arrow, center). Single confocal section. Scale bar = 5 μm. **(C)** Cells expressing GAD67-GFP and immunoreactive for PV were occasionally labeled by Retrobeads. Scale bar = 10 μm. **(D)** Cells immunoreactive for CB were occasionally labeled by Retrobeads. Scale bars = 10 μm. **(E)** Most cells in the hilus immunoreactive for CCK were also labeled by Retrobeads. Scale bar = 10 μm. **(F)** Cells in the hilus immunoreactive for GluA2 and labeled by Retrobeads were also observed. Scale bar = 20 μm.

To further examine which neurochemical hilar neuron types other than SOMIs project to the contralateral DG, we combined ipsilateral AAV-FLEX-GFP injection with retrograde tracing using red fluorescent Retrobeads (Ret) in SOM-cre mice (Figure 3), and confirmed that SOMIs making contralateral projections can be labeled using this method. As expected from the dense contralateral projection of glutamatergic, cholecystokinin (CCK)-expressing mossy cells, many Ret-positive (Ret^+^) cell bodies in the hilus were also immunopositive for CCK. In order to investigate contralateral projections made by other classes of interneuron, we unilaterally injected Ret into the hilus of GAD67-GFP mice and quantified the density of Ret^+^ cells co-expressing SOM, PV, calbindin (CB), calretinin (CR) or CCK. For the identification of putative mossy cells, we also applied antibodies against the ionotropic Glutamate receptor A2 subunit (GluA2; Leranth et al., 1996; Scharfman, 2016). We found a high density of Ret^+^ cells with large somata (∼14–24 μm diameter) co-expressing CCK (13769 cells/mm^3^; 94% of the putative mossy cell population) or GluA2 (14897 cells/mm^3^; 70% of the putative mossy cell population) but not GAD67-GFP (Figures 3E,F), indicating their mossy cell natures (Leranth et al., 1996; Scharfman, 2016). Indeed, this population formed the majority of retrogradely labeled cells, as few of the total Ret^+^ population co-expressed SOM (253 SOMIs/mm^3^; Figures 3A,B). Ret^+^ cells co-expressing PV (Figure 3C) showed similar densities (242 cells/mm^3^) to SOMIs, but those positive for CB (CBIs) were characterized by a markedly lower density (67 cells/mm^3^; Figure 3D). Although GCs express CB, they lacked Ret, further indicating that CB-labeling represented interneurons (CBIs).

From the entire interneuron population (19138 ± 3820 cells/mm^3^; see section Materials and Methods for definition), we estimated that 1.8% were SOMIs that formed projections to the contralateral hippocampus (253 out of 6344 SOMIs/mm^3^; 4.4% of all SOMIs). The relative fraction was lower for projecting PVIs (1.2% of all interneurons; 242 out of 3930 PVIs/mm^3^; 6.6% of all PVIs) and projecting CBIs (0.4% of all interneurons; 67 out of 467 CBIs/mm^3^; 16.7% of all CBIs) due to their smaller populations. Because SOM and PV are co-expressed in CA1 *oriens lacunosmum moleculare* (O-LM) interneurons (Freund and Buzsáki, 1996), we tested whether a similar co-expression profile may exist in the hilus. Interestingly, only 3.1% of SOMIs were also PV^+^ [12 out of 381 cells; in the reverse manner, this was 12 out of 222 PV^+^ cells (5.4%) that co-expressed SOM], suggesting that this cannot completely account for the entire population of projecting interneurons. We observed a strong immunolabeling for CR in small cell bodies located at the hilus-gcl border, an area containing adult-born GCs (Brandt et al., 2003; Spampanato et al., 2012). A very faint CR labeling was seen in larger multipolar cells in the hilus with the appearance of mossy cells (Liu et al., 1996; Blasco-Ibáñez and Freund, 1997). These cells were frequently and strongly Ret^+^, but never expressed GAD67-GFP (data not shown). Moreover, a diffuse but intense immunolabeling for CR was also observed in the inner molecular layer of the DG, which is known to be the axonal projection area of ipsi- and contralateral mossy cells (Liu et al., 1996; Scharfman, 2016). Based on these observations, we could not unequivocally identify CR^+^ GABAergic cells. Thus, although we noted a very small number of candidate CR^+^ interneurons that were also Ret^+^, we did not attempt to quantify CR^+^ cells further. In summary, hilar interneurons projecting to the contralateral DG are largely formed by SOMIs and PVIs.

### Contralaterally Projecting Interneurons Form Symmetric Synapses Onto GCs and Interneurons

**FIGURE 4:**
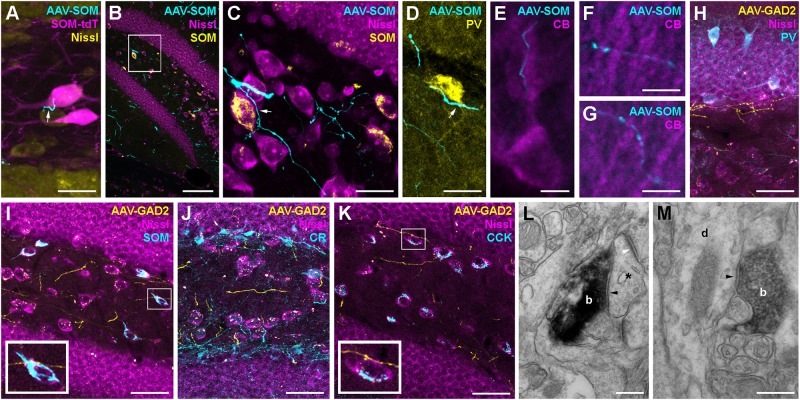
Hippocampal interneurons are likely to form synaptic connections with a diverse set of contralateral targets. **(A)** Contralateral axon from a SOM-cre GFP-virus-expressing neuron (cyan) forms a bouton (white arrow) in close proximity to a SOM-cre^∗^tdTomato-positive hilar interneuron (magenta). Maximum intensity projection of 6 confocal planes (1 μm spacing). Scale bar = 20 μm. **(B)** Low magnification image of the dentate gyrus contralateral to hilar virus injection in a SOM-cre mouse. Axons (cyan) can be seen in the hilus and the adjacent granule cell and molecular layers. Single confocal plane. Scale bar = 100 μm. **(C)** Enlarged view of the boxed region in **(B)**, showing a contralateral axon (cyan) in close apposition to a SOM-immunoreactive (yellow) interneuron cell body (arrow). Note that most axonal varicosities are not adjacent to any Nissl-labeled somata. Maximum intensity projection of 15 planes (1 μm spacing). Scale bar = 20 μm. **(D)** Contralateral axons from SOM-INs (cyan), one in close apposition to a PV-immunoreactive (yellow) soma at the hilus-GCL border (arrow). Maximum intensity projection of nine planes (1 μm spacing). Scale bar = 20 μm. **(E–G)** Contralateral axons from SOM-INs (cyan) in close apposition to CB-immunopositive Granule cell proximal, inner molecular layer **(E)** and distal, outer molecular layer **(F,G)** apical dendrites. Scale bars = 5 μm. **(H–K)** Low magnification images of the dentate gyrus contralateral to hilar virus injection in a GAD2-cre mouse co-labeled for PV **(H)**, SOM **(I)**, CR **(J),** or CCK **(K)**. Maximum intensity projections of 11, 8, 13, or 5 planes (1 μm spacing). Scale bars = 40 μm. Insets of boxed regions at 2.5x zoom highlight potential contact points with immunolabeled cells. Note that most axons are not adjacent to any visible structure. **(L)** Electron micrograph of a symmetric synapse (black arrowhead) formed by a contralateral axon terminal (b) of a GAD2-cre neuron onto a small postsynaptic profile (asterix) in the dentate molecular layer. Note the presence of an asymmetric, excitatory synapse onto the same dendritic profile (white arrowhead). Scale bar = 250 nm. **(M)** Electron micrograph of another symmetric synapse (arrowhead) formed by a virus-labeled GAD2-cre contralateral axon bouton (b) onto a dendritic profile (d) running radially in the dentate molecular layer. Scale bar = 250 nm.

Do contralaterally projecting interneurons form GABAergic synapses? To address this question, we performed high resolution confocal and electron microscopy (see section Materials and Methods). The postsynaptic targets of contralaterally projecting axons were identified using immunolabeled sections from AAV-injected SOM-cre, SOM-tdT and GAD2-cre mice. Sections were carefully examined for close appositions between GFP-expressing, contralaterally projecting axons and cell somata or processes immunoreactive for neurochemical markers including SOM, PV, CB, CCK, and CR (Figure 4). Confocal image stacks of contralaterally projecting axons from SOMIs in SOM-cre mice occasionally formed varicosities in close apposition to SOM-expressing somata identified either in SOM-tdT mice (Figure 4A), or upon antibody labeling against SOM in SOM-cre mice (Figures 4B,C). Moreover, SOM axons were further observed in close proximity to PV^+^ somata at the hilus-gcl border (Figure 4D), and to proximal and distal dendrites of CB^+^ GCs of the contralateral DG (Figures 4E,G). Unilateral injection of AAV-FLEX-GFP in GAD2-cre mice resulted in the labeling of axons in the contralateral DG with one or two varicosities in close apposition to SOMIs (Figure 4I, inset) and CCK-expressing mossy cells (Figure 4K, inset). However, most contralateral axons in any given region of the DG did not appear to be adjacent to any labeled neuronal structure (Figures 4B–K).

Pre-embedding immunohistochemistry for GFP in samples from GAD2-cre mice injected with AAV-FLEX-GFP labeled contralateral axons in the DG molecular layer and hilus. Further processing of the samples for DAB staining and subsequent electron microscopy revealed that putative synapses were formed in the molecular layer of the contralateral DG (Figures 4L,M). They contained flattened vesicles and made symmetric appositions with a clear widening at the synaptic cleft (Figures 4L,M; black arrowheads). The postsynaptic profiles were either small compartments that received asymmetric, excitatory synaptic inputs from non-labeled axons (Figure 4L, white arrowhead), or larger dendritic profiles that ran perpendicular to the gcl (Figure 4M), and were likely to be GC dendrites. Thus, our data indicate that contralateral axons from interneurons are likely to form functional GABAergic contacts onto primarily distal dendrites of neurons, very likely GCs, as well as hilar neurons, including mossy cells and several interneuron subtypes, including SOMIs and PVIs.

**FIGURE 5:**
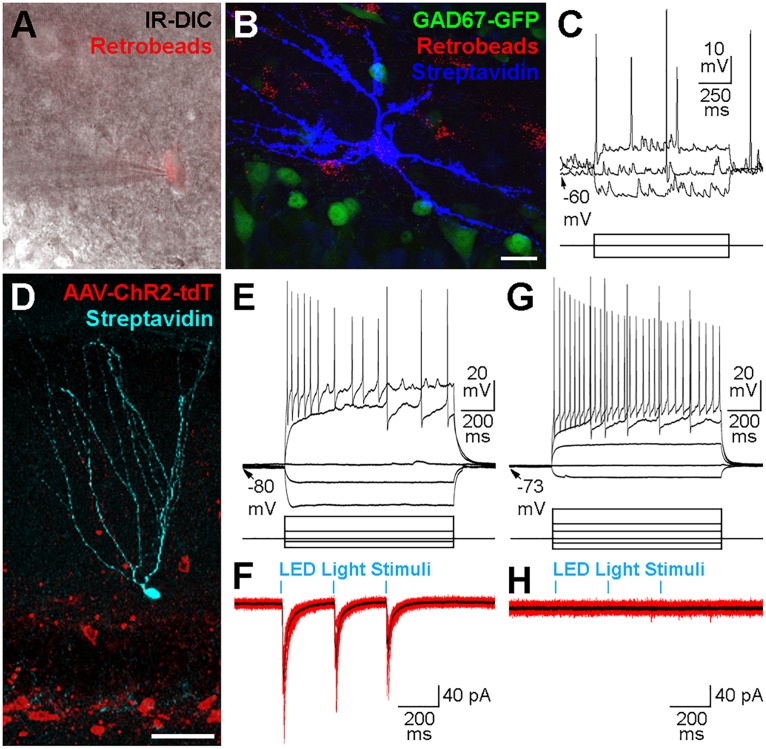
Physiological properties of contralateral projections. **(A)** Cells containing Retrobeads were targeted for whole-cell patch-clamp recordings using infra-red differential interference contrast microscopy (IR-DIC). **(B)** The same cell in **(A)** after visualization with Alexa 647-conjugated Streptavidin (blue) did not express GAD67-GFP (green). Maximum intensity projection of 42 planes at 1 μm intervals. Scale bar = 20 μm. **(C)** Voltage responses to current injections of the cell shown in **(A,B)**. Scale bars = 10 mV or 175 pA, 250 ms. **(D)** Confocal image of a granule cell recorded, filled with biocytin and visualized *post hoc* with Alexa647-conjugated Straptavidin (cyan). The slice was from the ipsilateral hippocampus of a GAD2-cre mouse 3 weeks after a hilar injection of an AAV expressing Channelrhodopsin2 and tdTomato (red) in a cre-dependent manner. Maximum intensity projection of 23 planes at 2 μm intervals. Scale bar = 50 μm. **(E)** Voltage responses to current injections of the cell shown in **(D)**. Scale bars = 20 mV or 200 pA, 200 ms. **(F)** Current responses (10 individual overlaid sweeps, red; average, black) from the granule cell shown in **(D,E)** to blue LED light pulses (5 ms duration, 250 ms apart). Scale bar = 40 pA, 200 ms. **(G)** Voltage responses to current injections of a different Granule cell in a slice contralateral to the hilar injection of AAV-Flex-ChR2-tdT in a GAD2-cre mouse. Scale bars = 20 mV or 200 pA, 200 ms. **(H)** Current responses (10 individual overlaid sweeps, red; average, black) were not observed from the same Granule cell presented in **(G)** to blue LED light pulses (5 ms duration, 250 ms apart). Scale bar = 40 pA, 200 ms.

We used the whole-cell patch clamp technique in acute hippocampal slice preparations to record and intracellularly fill contralateral Ret^+^ hilar cells (Figure 5A) with biocytin. From 19 successfully recorded cells from dorsal coronal hippocampal slices made from 4 injected mice, 7 cells had morphological and physiological attributes consistent with mossy cells (Figures 5B,C). However, we did not successfully locate, record and recover cells with interneuron morphologies and sufficient axon to identify their sub-type (e.g., HIPP, HIL, MOPP etc.). To examine whether contralaterally projecting interneurons release GABA, we performed whole-cell patch clamp recordings from potential post-synaptic target cells in combination with optogenetic recruitment of contralaterally projecting GABAergic axons. For these experiments we stereotaxically injected AAVs encoding Channelrhodopsin2 (ChR2) and tdTomat in a Cre-recombinase-dependent manner (AAV-Flex-ChR2-tdTomato) unilaterally in the DG of 7 GAD2-cre mice (Figures 5D–H). ChR2-expressing axonal projections were predominantly observed in the molecular layer (see also Figures 4E–G). We therefore targeted GCs for whole-cell recordings (Figure 5D). In the ipsilateral DG, light pulses (three pulses, 4 Hz, 5 ms pulse duration, 100% of 493 nm LED intensity) evoked large-amplitude IPSCs in GCs with high probability (Figures 5D–F). However, light-mediated signals could not be evoked in any of the 25 cells (GCs or interneurons) recorded in slices made from the contralateral hippocampus (Figures 5G,H). This finding was also true when applying five pulses at 40 Hz (5 ms pulse duration, 100% LED intensity; data not shown), suggesting that this activity pattern was also insufficient to drive detectable responses in potential target cells, probably due to the low density of contralateral axonal fibers and synaptic contact sites.

### Monosynaptic Retrograde Rabies Virus Tracing Labels SOMIs and Mossy Cells

**FIGURE 6:**
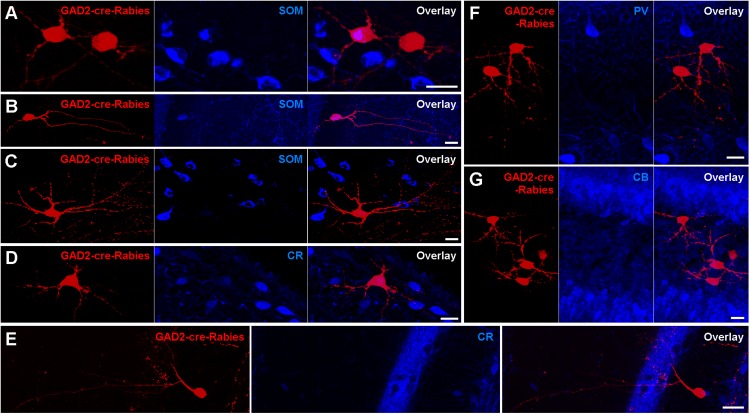
Retrograde *trans*-synaptic tracing with modified Rabies virus indicates Mossy cells as the main contralateral presynaptic partners of Hilar interneurons. **(A,B)** Occasional neurons in the contralateral hilus expressing the G-deleted Rabies virus (red) are also immunoreactive for SOM (blue). Starter cells were restricted to ipsilateral GAD2-cre-expressing hilar neurons (not shown). Maximum intensity projection of 36 and 27 planes (1 μm spacing). Scale bars = 20 μm. **(C,D)** Neurons in the contralateral hilus with mossy cell morphologies immunonegative for SOM **(C)** and CR **(D)**. Maximum intensity projection of 41 and 16 planes (1 μm spacing). Scale bars = 20 μm. **(E)** A neuron with interneuron-like morphology immunonegative for CR. Maximum intensity projection of 15 planes (1 μm spacing). Scale bar = 20 μm. **(F,G)** Clusters of neurons in the contralateral hilus with mossy cell morphologies that were immunonegative for PV **(F)** or CB **(G)**. Maximum intensity projection of 32 and 46 planes (1 μm spacing). Scale bar = 20 μm.

To examine the nature of presynaptic neurons targeting contralaterally projecting interneurons, we applied rabies virus (RABV)-based monosynaptic tracing (Figure 6). Unilateral AAV infection in the DG of GAD2-cre mice induced a Cre-dependent expression of GFP, Protein G and the hTVA receptor in GAD2-cre-expressing cells, and thereby primed these cells for Rabies infection. Twenty-two days later an EnvA-serotyped, G-deleted RABV was injected at the same location, and we then waited a further 17 days, during which time the RABV jumped to presynaptic neurons and induced the expression of mCherry only. Thus, ‘primed’ cells express only GFP, ‘starter’ cells express both GFP and mCherry, whereas presynaptic cells express only mCherry. In principle, the RABV particles from original starter cells could result in mCherry expression in their presynaptic ‘primed’ cells. However, arguing against this, we observed very few starter cells (∼2 per section) in the injected hilus. In contrast, there were many mCherry^+^ presynaptic cells in the same hippocampal hemisphere, such as GCs, mossy cells, cells with interneuron-like morphologies and CA3 cells, including PCs. In the contralateral hippocampus, mCherry^+^ neurons were located mostly in the hilus. The majority of mCherry^+^ cells had mossy-cell-like morphologies (30/50 cells had large, triangular somata and thorny excrescence-bearing dendrites confined to the hilus) and were SOM^–^ (Figure 6C). Moreover, ∼6% of mCherry^+^ cells expressed SOM (Figures 6A,B), ∼4% CR (Figure 6D), and none of the labeled cells co-expressed PV or CB (Figures 6F,G). These data are in line with our GFP-expression and retrograde tracing data, and further show that among interneuron types, SOMIs in particular contact contralateral GABAergic cells.

## Discussion

Here we show that genetically defined SOMIs in the DG-hilus, CA3 and CA1 hippocampal regions make topographic axonal projections to GC, GABAergic cell and mossy cell targets in the contralateral hippocampus, and predominantly target their distal dendrites. We numerically quantify these connections in terms of projecting cell numbers and the density of their axonal ramification in each region of the hippocampus, and show that these axons have a mean density of ∼10% of their ipsilateral counterparts. We show that other neurochemically defined interneuron types, such as PVIs, also participate in these contralateral projections, but make much smaller contralateral axon ramifications. We show that the axonal projections made by SOMIs appear to be topographic, with cells in each area innervating their corresponding contralateral area, whereas PVIs do not seem to follow this pattern. Our monosynaptic rabies tracing data also indicate that the majority of presynaptic inputs to hilar interneurons arise from hilar mossy cells, but that there are also interneuron-to-interneuron connections from contralateral SOMIs.

### The Extent of the Commissural Projection Made by Interneurons

Previous studies have demonstrated that the commissural projection between hippocampi mainly consists of hilar mossy cell axon collaterals that ramify in the inner molecular layer (Scharfman, 2016). A few studies, using retrograde tracers in conjunction with fluorescent immunocytochemistry, have further shown that a small fraction of commissural projection cells express GAD (Ribak et al., 1986), SOM (Léránth and Frotscher, 1987), NPY (Blasco-Ibáñez and Freund, 1997), or PV (Goodman and Sloviter, 1992). Previous estimates of the contralaterally projecting SOMIs (1% of all retrograde-labeled cells, Bakst et al., 1986; 2.3%, Blasco-Ibáñez and Freund, 1997), the NPY-expressing population (3.6%, Blasco-Ibáñez and Freund, 1997) or the PVI population (1%; Goodman and Sloviter, 1992) are in close agreement with our data using Retrobead labeling (1.6%). Also similar to these reports, we found fewer Ret^+^ interneurons in the contralateral ventral hilus, even when we injected in this region ipsilaterally, and found an even sparser commissural axonal projection from SOMIs in this region. We typically injected relatively large amounts of Retrobeads (500–1000 nl) in order to label as much of the projecting population as possible, and this was confirmed by the 24-fold larger number of Ret^+^ mossy cells compared to Ret^+^ interneurons. There might be a cell-type specific preferential uptake of Retrobeads, such that interneurons are seldom labeled. However, we saw similar results with other retrograde tracers such as the Cholera Toxin Beta subunit, Microruby (fluorescently conjugated dextran) and canine adeno-virus expressing mCherry (data not shown). These data complement similar methods used in the literature, such as Fluorogold (Goodman and Sloviter, 1992; Zappone and Sloviter, 2001), lipophilic dyes (Swanson et al., 1981) or horseradish peroxidase (Ribak et al., 1985). Thus, we conclude that compared to the mossy cell population, the number of contralaterally projecting interneurons appears to be small, and consists of mainly SOMIs and PVIs.

When we compared the patterns of contralateral axonal ramification made by genetically defined interneurons in GAD2-cre and SOM-cre mice, we found that they were highly similar. Because GAD2 is expressed in almost all DG-interneurons (Wang et al., 2014), this indicates that the majority of the contralateral axons that we observed are likely to arise from SOMIs. This conclusion is supported by AAV injections in PV-cre mice, which demonstrated a much lower density of contralateral axons compared to their SOMI counterparts. Furthermore, the ramification pattern was fundamentally different, despite a similar viral infection of the DG and CA3 (Figures 2C,F). The overlap between SOM and PV expression in neurons of the DG that we measured (2%) is smaller than the fraction of interneurons that make contralateral projections, suggesting that this colocalization cannot be used to define this sub-population. Given the differences in the sub-regions targeted by contralateral axons of the two interneuron types, we propose that the SOMIs and PVIs making contralateral projections are likely to originate from two different populations. This is further supported by the differences in contralateral axon density observed in PV-cre and SOM-cre mice injected with AAV. Our data from PV-cre mice are in line with what might be expected from a low number of cells traced by Retrobeads. In contrast, despite their low abundance, SOMIs made a disproportionately large contralateral projection, with much higher axon densities, and a clearer topographic ‘same-region-to-same-region’ pattern of innervation than PVIs. Differences in contralateral projections between PVIs and SOMIs cannot be explained by differences in the number of labeled somata on the ipsilateral hemisphere because we injected similar volumes of AAV, and it appeared that we infected most, if not all, SOMIs or PVIs within the infected region. GFP expression was strong in the infected cells, consistent with only one or a few cre molecules being required for recombination of the injected AAV genome. Although we cannot completely rule out the possibility, we did not observe any viral tropism in favor of particular neuronal subtypes, and the AAV seemed able to infect neurons and generate GFP expression in all the cre-expressing lines we tested (including other lines not reported in this manuscript). Thus, the differences in contralateral innervation that we have observed seem to be dependent upon the interneuron type.

### Which Interneuron Types Make Contralateral Projections?

Interneurons are very diverse, with different types serving different functional roles, as indicated by their axonal distributions and synapse locations at defined compartments of principal cells (Han et al., 1993; Freund and Buzsáki, 1996). Fast-spiking PVIs comprise basket cells and axo-axonic cells, and one possibility is that a sub-population of these perisomatic-targeting PVIs make sparse contralateral projections to (predominantly) CA1 (Figure 2F). Alternatively, a small, distinct PVI subpopulation may be the origin of the PVI contralateral projections, which may also express additional neurochemical markers (such as vasoactive intestinal polypeptide, CB or CR), but this remains to be determined. SOMIs are a very diverse population including hilar perforant path-associated cells (HIPPs), total molecular layer (TML) and hilar interneurons (HILs) (Freund and Buzsáki, 1996; Hosp et al., 2014; Savanthrapadian et al., 2014; Yuan et al., 2017). HILs are known to make long-range projections to the medial septum, primarily inhibiting glutamatergic cells (Yuan et al., 2017). Thus, this SOMI subtype is a potential candidate for the source of SOMI contralateral projections. However, we observed a greater axonal density in the ipsi- and contralateral DG-molecular layer (Figure 2 and Table 1) and found evidence for interneurons targeting contralateral apical GC dendrites (Figure 4), suggesting that other SOMI subtypes forming axonal projections in the molecular layer, such as HIPP and TML cells, may also target the contralateral molecular layer.

Previous literature indicates that interneurons in CA1 that project axons to other regions express a diverse range of neurochemical markers. Examples include muscarinic M2 receptor-expressing cells in all strata of CA1 projecting to the retrosplenial cortex (Miyashita and Rockland, 2007), stratum radiatum cells expressing enkephalin (Fuentealba et al., 2008), stratum oriens cells expressing vasoactive intestinal polypeptide (Francavilla et al., 2018) that project to the subiculum, and stratum radiatum – lacunosum-moleculare border cells that express COUP-TFII and project into the DG molecular layer (Fuentealba et al., 2010). Although not specifically tested for SOM expression, some of these cells express Neuropeptide Y, and nNOS, which are often co-expressed in SOMIs. Indeed, Sik et al. (1994) report that cells in CA1 stratum oriens express NADPH and are likely to be SOMIs. Our quantitative analysis of injections into CA1 in SOM-cre mice detected a small innervation of ipsilateral CA3 and DG (Table 1), which may be accounted for by GFP expression in such ‘retro-hippocampal’ cells (Sik et al., 1994). Given the relative sparsity of the contralateral projections to contralateral CA1, we consider it likely that ‘retro-hippocampal’ cells, hippocampal-septal projection cells in stratum oriens (Gulyas et al., 2003; Takács et al., 2008) or a completely different interneuron category are responsible, rather than the much more common Oriens-Lacunosum-Moleculare or Oriens-Bistratified cells types of CA1, although this possibility cannot be totally excluded.

### What Is the Potential Function of Contralaterally Projecting Interneurons?

We attempted to investigate the potential functional role of contralaterally projecting interneurons (Figure 5), but they appeared to be particularly vulnerable to the slicing procedure, as has also been observed for hippocampal-septal cells in CA1 (Gulyas et al., 2003). Furthermore, the sparseness of the connections and their likely location on GC and interneuron distal dendrites (Buckmaster et al., 2002) made it challenging to detect synaptic currents. Indeed, it has been shown that ‘ipsilateral’ DG SOMI functional connectivity is sparse (Espinoza et al., 2018). However, we propose that the contralateral projections provide feed-forward inhibition and thereby complement the feed-forward excitation of the commissural mossy cell projections onto GC and interneuron apical dendrites. Alternatively, by providing predominantly feed-forward inhibition onto DG-interneurons, contralaterally projecting SOMIs could function as a disinhibitory gate, similar to observations from SOMIs in the lateral entorhinal cortex projecting to CA1 (Basu et al., 2016). Hilar SOMIs do not respond to entorhinal inputs when using stimulation strengths that effectively activate most other interneurons (Lee et al., 2016), suggesting that they may be recruited by other sources or under special conditions (Stefanelli et al., 2016; Urban-Ciecko and Barth, 2016). Indeed, timing in the circuit may be critical, as the extra synaptic delay caused by routing through an interneuron connection may be important for such functions as phase resetting. GABAergic cells play a key role in the synchronization of principal cells and the generation of fast brain rhythms (Bartos et al., 2007). Over a few oscillatory cycles, activity of SOMIs could synchronize and transmit rhythmic activity from the ipsi- to the contralateral side. Sparse contralateral inputs to the whole network (via ‘hub’ neurons), thus may support synchronicity of rhythmic activity of the two hippocampal hemispheres when memory processing demands are high. A similar role for CA1 interneurons in synchronizing, rather than strongly inhibiting, the subiculum has recently been proposed (Francavilla et al., 2018). Future investigations using *in vivo* recording techniques will be required for a complete axo-dendritic labeling of these cells in order to address these challenging questions.

## Data Availability

The raw data supporting the conclusions of this manuscript will be made available by the authors, without undue reservation, to any qualified researcher.

## Ethics Statement

Animal Subjects: The animal study was reviewed and approved by the University of Freiburg and the State of Baden-Württemberg (Regierungspräsidium Freiburg), Germany.

## Author Contributions

ME and MB designed the study. ME conducted the experiments and performed the statistical analysis. ME and MB wrote the manuscript and approved the submitted version.

## Conflict of Interest Statement

The authors declare that the research was conducted in the absence of any commercial or financial relationships that could be construed as a potential conflict of interest.

## Footnotes

1 www.lumafluor.com
