# Supplementary figures and images for: Somatostatin-Expressing Interneurons Form Axonal Projections to the Contralateral Hippocampus
Source: Front Neural Circuits. 2019 Aug 23;13:56. doi: 10.3389/fncir.2019.00056 (PMC6716454; doi:10.3389/fncir.2019.00056)

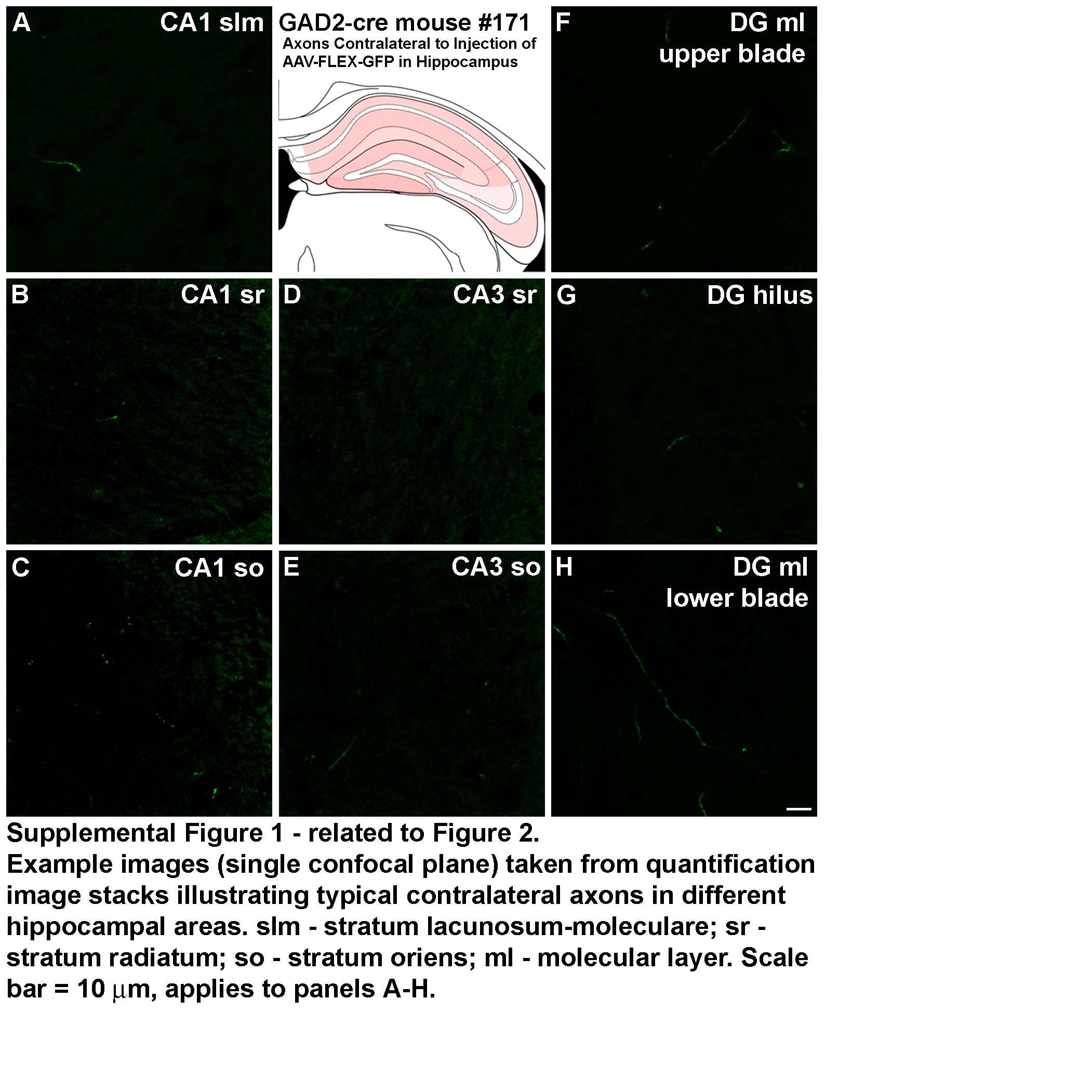

Supplement: Supplementary file 1 [file Image_1.TIF]
