# Supplementary material for: Somatostatin-Expressing Interneurons Form Axonal Projections to the Contralateral Hippocampus
Source: Front Neural Circuits. 2019 Aug 23;13:56. doi: 10.3389/fncir.2019.00056 (PMC6716454; doi:10.3389/fncir.2019.00056)
Supplement: Supplementary file 2 [file Table_1.docx]

**SUPPLEMENTARY TABLE 1 | Stereotaxic co-ordinates and related data for the injections in this study.**

| Experiment | Genotype | No. of mice | Age at injection (days) | Survival (days) | Target area | Posterior from Bregma (mm) | Lateral from Bregma (mm) | Depth from pia (mm) | Volume injected (nano-liters) | Substance Injected |
| --- | --- | --- | --- | --- | --- | --- | --- | --- | --- | --- |
| Axonal quantification | GAD2-cre | 3 | p81-94 | 18-34 | hippo-campus | -2.200 | ±1.350 | -1.750 | 1000 | AAV2/1-CAG-FLEX-GFP |
| Axonal quantification | SOM-cre | 3 | p78, 344, 350 | 17-29 | hippo-campus | -2.400 | ±1.350 | -1.750 | 1000 | AAV2/1-CAG-FLEX-GFP |
| Axonal quantification | SOM-cre | 4 | p45-102 | 17-22 | hilus | -2.000 | ±1.360 – 1.470 | -1.750 | 1000 | AAV2/1-CAG-FLEX-GFP |
| Axonal quantification | SOM-cre | 4 | p63-78 | 17-20 | hilus and CA3 | -2.000 | ±2.500 | -1.950 | 850 | AAV2/1-CAG-FLEX-GFP |
| Axonal quantification | SOM-cre | 4 | p78 | 18-21 | CA1 | -2.000 | ±1.350 | -1.300 | 500 | AAV2/1-CAG-FLEX-GFP |
| Axonal quantification | PV-cre | 4 | p77 | 27 | hilus and CA3 | -2.000 | ±1.370 | -1.750 | 1000 | AAV2/1-CAG-FLEX-GFP |
| Retrobeads | GAD67-GFP | 3 | p89-119 | 14-15 | hilus | -2.000 | ±1.330-1.470 | -1.750 | 1000 | Undiluted Retrobeads |
| In vitro slices | GAD67-GFP | 4 | p49 | 1-6 | hilus | -2.000 | ±1.370 | -1.750 | 200 | Undiluted Retrobeads |
| ChR2 | GAD2-cre | 7 | p71-73 | 14-25 | hilus | -2.000 | ±1.370 | -1.750 | 400-1000 | AAV-CAG-FLEX-ChR2-YFP |
| Retrograde tracing | GAD67-GFP | 5 | p88-108 | 3-8 | hilus | -2.000 | ±1.370 | -1.750 | 250-1000 | 1% CTB dissolved in PBS |
| Mono-synaptic Rabies tracing | GAD2-cre | 5 | p57-93  p79-115 | 19-27  15-25 | hilus | -2.000 | ±1.370 | -1.750 | 500-750  500-1000 | AAV-EF1a-FLEX-GFP-hTVA  EnvA-delta-G-Rabies-mCherry |
